# Cellular Alterations in Carbohydrate and Lipid Metabolism Due to Interactions with Nanomaterials

**DOI:** 10.3390/jfb14050274

**Published:** 2023-05-14

**Authors:** Ana Martín-Pardillos, Pilar Martin-Duque

**Affiliations:** 1 Instituto de Nanociencia y Materiales de Aragón (INMA), CSIC-Universidad de Zaragoza, 50009 Zaragoza, Spain; 2 Department of Chemical Engineering and Environmental Technology (IQTMA), University of Zaragoza, 50018 Zaragoza, Spain; 3 Instituto de Investigaciones Sanitarias de Aragón (IIS Aragón), 50009 Zaragoza, Spain; 4 Ciber Bioingeniería y Biomateriales (CIBER-BBN), Instituto de Salud Carlos lll, 28029 Madrid, Spain; 5 Surgery Department, Medicine Medical School, University of Zaragoza, 50009 Zaragoza, Spain

**Keywords:** nanoparticle, metabolism, lipid, carbohydrate, diabetes, obesity, cancer, oxidative stress, inflammation

## Abstract

Nanoparticles (NPs) have unique physicochemical properties that are useful for a broad range of biomedical and industrial applications; nevertheless, increasing concern exists about their biosafety. This review aims to focus on the implications of nanoparticles in cellular metabolism and their outcomes. In particular, some NPs have the ability to modify glucose and lipid metabolism, and this feature is especially interesting to treat diabetes and obesity and to target cancer cells. However, the lack of specificity to reach target cells and the toxicological evaluation of nontargeted cells can potentially induce detrimental side effects, closely related to inflammation and oxidative stress. Therefore, identifying the metabolic alterations caused by NPs, independent of their application, is highly needed. To our knowledge, this increase would lead to the improvement and safer use with a reduced toxicity, increasing the number of available NPs for diagnosis and treatment of human diseases.

## 1. Introduction

### 1.1. Nanomaterial Definition and Types of Classifications Regarding Their Composition

Studies on nanostructured materials (NSMs) are research areas due to their application in many domains [1]. NSMs are materials with sizes that range from 1 to 1000 nm, at least one dimension; nonetheless, they used to be classified in a range from 1 to 100 nm. Nanoparticles (NPs) are nano-objects with three external nanoscale dimensions, which have shown great potential in biological and biomedical applications because of their distinct physical and chemical properties [2]. However, their biosafety is raising concerns worldwide and must be evaluated comprehensively before their clinical applications [2,3].

NSMs can be classified according to their origin, dimensionality, morphology, composition, uniformity, and agglomeration (Table 1) [1,4,5]. We can divide NSMs according to different features that are highly relevant and can be determining for the toxicity of these compounds:

- Based on their origin, NSMs can be natural or synthetic (anthropogenic nanomaterials) [4,5,6,7,8,9,10].
- Although the first classification of nanostructured materials (NSMs) was postulated according to their chemical composition and the dimensionality (shape) [11], the creation of novel nanostructures made it necessary to reformulate this classification [12] considering the electron movement along the dimensions [1,13].
- Morphology is defined by flatness, sphericity, and aspect ratio [1,4]. The main phenomena of NSMs occur at the surface, meaning that the total surface should be considered in terms of toxicology [14]. The aspect ratio determines the internalization rate of a particle, and as the aspect ratio increases, toxicity increases as well [15]. Nanoparticles defined as having a high aspect ratio include nanotubes and nanowires, varying in shape and length. Meanwhile, nanoparticles defined as having a small aspect ratio include spheres, ovals, cubes, prisms, helixes, and pillars [4].
- NSMs can be constituted by a single material or a mixture of various materials [4]. Most current NSMs are divided into four categories according to their material composition: carbon-based nanomaterials [8,9,16], inorganic-based nanomaterials (metal and metal oxide), organic-based nanomaterials [17], and composite-based nanomaterials [7,18].
- NSMs can exist as dispersed or agglomerated aerosols, and the surface properties primarily determine the agglomeration state and uniformity of the particles closely related to their activity and toxicity [4,19,20].

All these aspects could be crucial for NSM interaction with cells, including all the changes produced in their metabolism, which we will study in this review and will be included in Table 1.

### 1.2. Nanomaterials and Their Classification Regarding Their Applications in Medicine

NPs are very diverse and have been used in many medical applications, including diagnosis, treatment, and both at the same time (theragnosis).

#### 1.2.1. NPs as Drug Delivery Systems

NPs have been used in the clinic setting as drug delivery systems since the early 1990s [21]. Among the NP formulations designed and tested in recent years, we find gold nanoparticles [22], ultrasmall superparamagnetic iron oxide (USPIO) nanoparticles [23], nanotubes [24], and nanoparticles encapsulated in liposomes [25], polymers, and micelles [26,27].

Regarding their size, it has been accepted that the optimal size for drug delivery systems ranges between 10 and 100 nm [28]. Particles with a size lower than 5 nm are quickly eliminated from the bloodstream through extravasation or renal clearance; as the particle size increases to more than 150 nm, nanoparticles are preferably accumulated in the liver, spleen, and bone marrow [29,30].

Particle shape is equally important to size to determine NP endocytosis [30,31,32,33]. The improvement of NP pharmacodynamics can be achieved by modifying their surfaces with biocompatible polymers and proteins, which result in improved colloidal stability, sustained blood circulation, and decreased toxicity [10,34]. For example, the tendency of the nanoparticles to be accumulated in the “reticuloendothelial system” (RES) is a consequence of the adsorption of specific proteins on the surface of the nanoparticles [29,35,36]. The most common modification to reduce uptake by the RES and extend circulation lifetime is by PEGylation (PEG refers to PolyEthylene Glycol) [37,38]. Recently, other modifications, such as lactose, have been tested to avoid immune responses to PEG, clearance on repeat injection, and anaphylactic reactions. The results show even reduced levels of inflammatory cytokines, thereby extending circulation half-life and enhancing delivery to tumors and other organs [39]. Changing the NP surface for their optimal internalization could be performed by modifying the external charge, and for that purpose, chitosan has been widely used [40,41].

Additionally, in order to target cells and their microenvironment with high specificity and affinity and to increase the concentration of drugs in selected target tissues and lessen systemic secondary and toxic effects, NPs are conjugated with ligands. Among these ligands, antibodies, aptamers, or small molecules are the most common [29,30,35,42,43]. Functionalized NPs could also be candidates to substitute viral vectors in the transfection of different cell lines [44]; among the most recent successful examples related to viruses are two mRNA-based coronavirus infectious disease (COVID-19) vaccines delivered by PEGylated lipid nanoparticles exist [38].

#### 1.2.2. NPs as Diagnosis Systems

Currently, most clinically approved NPs have therapeutic aims, despite their potential application in diagnosis. Up to now, just one nanoparticle has been approved for diagnosis, iron oxide nanoparticles [23]. The translation of nanoparticles from research to their use in clinical diagnosis is the consequence of several limiting factors. The features that make NPs good candidates for drug delivery are the same that complicate their use in diagnosis. While therapeutic NPs should have pharmacological activity, diagnostic NPs should not induce any physiological alteration, and they should be cleared after their detection [28]. Nevertheless, they have some features that make them promising candidates for diagnosis, such as remarkable optical, electronic, and magnetic features, depending on their composition. In addition, a broad range of receptors can be bonded to NPs, achieving affinities and selectivities, which allow them to target specific analytes. The combination of these features can be employed to perform the transduction of the binding events [45].

## 2. Effect of NPs on Cell Metabolism and Toxicity

Nanoparticles (NPs) can interact with biomolecules because of their large surface-to-mass ratio; this feature is useful for a broad range of biomedical applications; however, there is increasing concern about their biosafety [36,46]. Cells are constituted by water, inorganic ions, and organic molecules. Remarkably, the unique cellular constituents are the organic molecules. Organic compounds are divided into four classes of molecules: carbohydrates, lipids, proteins, and nucleic acids. Therefore, the cellular chemistry is defined by the structure and function of the basic classes of the organic molecules: carbohydrates, lipids, nucleic acids, and proteins [47]. The biochemical process that metabolizes nutrients and endogenous molecules to obtain energy and matter, as proteins, nucleic acids, and lipids that support life is called cellular metabolism [48,49]. Here, we will analyze the interactions of nanoparticles with carbohydrates and lipids, components involved in the cell/organism metabolism.

### 2.1. Implications in Carbohydrate Metabolism

Carbohydrates are constituted by simple sugars and polysaccharides. Among simple sugars, we find glucose, the primary nutrient of cells. Its metabolism results in a source of cellular energy and the initial material for the anabolism of other cellular components. Polysaccharides are involved in different processes, energy storage, structural components of the cell, recognition processes, and transport of molecules [47].

In this work, changes in carbohydrate metabolism will be analyzed from the perspective of diabetes and cancer.

#### 2.1.1. NP Reprogramming of Carbohydrate Metabolism in Cancer

Normal cells generate energy, adenosine 5′-triphosphate (ATP), through mitochondrial oxidative phosphorylation. Cancer cells undergo faster metabolism and consume significantly more glucose than normal cells, relying primarily on aerobic glycolysis; this phenomenon is called “the Warburg effect” [50,51]. This difference in carbohydrate metabolism to generate energy opens new opportunities to specifically target tumor cells while reducing the side effects on normal cells.

##### Beneficial Effect of NP Direct Reprogramming of Carbohydrate Metabolism for Cancer Treatment

**Figure 1:**
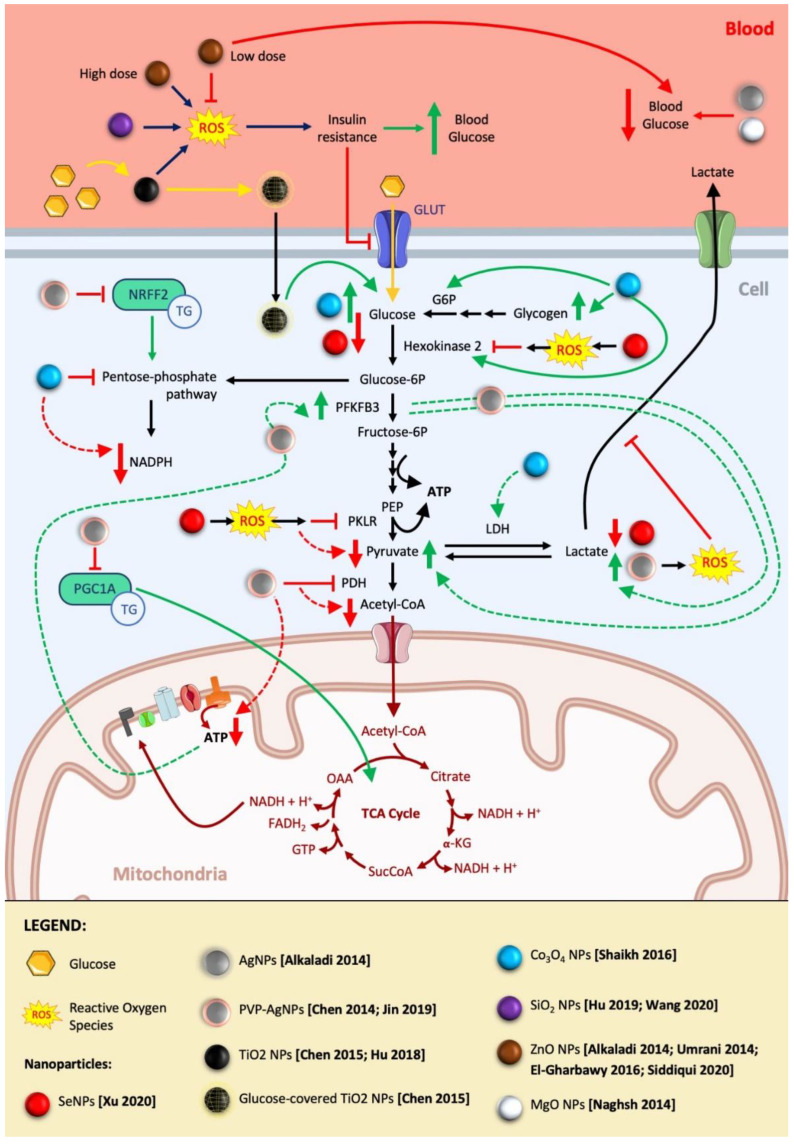
Effect of inorganic NPs in carbohydrate metabolism. Role of NPs in glycemia, glycolysis, and Krebs cycle. Red lines are negative stimuli (decrease in metabolite or enzyme activity), and green lines are positive stimuli (increase in metabolite or enzyme activity). Figure was partly generated using Servier Medical Art, provided by Servier, licensed under a Creative Commons Attribution 3.0 unported license [52,53,54,66,67,68,69,70,71,75,76,80,81,82].

A good example of NPs able to reprogram carbohydrate metabolism is silver nanoparticles (AgNPs). Treatment with AgNPs coated with polyvinylpyrrolidone (PVP) under sublethal concentrations, on a panel of human tumor cell lines with differential respiration rate, including human cervix cancer cell line HeLa, human prostate cancer cell line PC3, human hepatic carcinoma cell line HepG2, and human renal carcinoma cell line A498, increased the concentration of pyruvate and lactate and decreased the production of ATP in a dose-dependent manner (Figure 1) [52].

In the work, the authors described mechanisms by which PVP-AgNPs reprogram the energy metabolism as AgNPs disrupt several metabolism-related genes. Among them, they suppress peroxisome proliferator-activated receptor γ coactivator 1alpha (PGC-1a) decreasing the Krebs cycle and lipid metabolism; downregulate pyruvate dehydrogenase (PDH), inhibiting the metabolism of pyruvate to acetyl-coA and consequently reducing the initial substrate of the Krebs cycle; and downregulate oxidative phosphorylation-related genes, inhibiting the mitochondrial oxidative phosphorylation. These alterations compel the cells to modify the energy metabolism, increasing glycolysis through the upregulation of PFKFB3 (6-Phosphofructo-2-Kinase/Fructose-2,6-Biphosphatase 3) to compensate the reduction in ATP generation and cover the energy requirements for cell survival. The result of these alterations is an increase in pyruvate and lactate concentrations and a decrease in ATP production (Figure 1) [52].

As we previously mentioned, particle shape and size can determine NP endocytosis and, as consequence, their effect [30,31,32,33]. AgNPs from 20 to 45 nm with spherical and plate-like shapes show similar alterations of the energetical metabolism of the cell, as the decrease in ATP and increase in pyruvate and lactate levels reveal; however, smaller AgNPs induce a stronger effect, probably due to their higher tendency to enter the cells [52].

Similarly, using PVP-AgNPs reveals size-dependent effects in HepG2 cells. PVP-AgNPs measuring 5 nm (0.5–7.5 μg/mL) but not 100 nm reduce glucose consumption in a dose-dependent manner. However, a tendency of an increasing PFKFB3 protein level was detected in the HepG2 and Huh7 cell lines. On the other hand, in both cell lines, PVP-AgNPs induced a downregulation of Nuclear factor erythroid 2-related factor 2 (Nrf2), a regulator of the pentose phosphate pathway (PPP), a metabolic pathway parallel to glycolysis (Figure 1) [53].

##### Beneficial Effect of Indirect Reprogramming of Carbohydrate Metabolism for Cancer Treatment by Nanoparticle Alteration of Oxidative State

Metabolic reprogramming of tumor cells by ROS production has emerged as a new therapeutic strategy, and many research groups are trying to exploit it to kill cancer cells.

It has been shown how glioma cells treated with selenium NPs (SeNPs) inhibit the glucometabolic pathway in a ROS pathway-dependent manner, inducing apoptosis of cancer cells. This effect is a consequence of the reduction in glucose uptake, lactate production, and ATP levels, together with the downregulation of a series of critical glucose metabolism enzymes (Henokinase-2 and Pyruvate Kinase L/R (PKLR)) (Figure 1) [54].

However, not only SeNPs are able to modify the cell metabolism in an ROS-dependent manner. AgNPs are also capable to alter metabolic activity including carbohydrate metabolism, because of the increased generation of reactive oxygen species (ROS) [55]. Nevertheless, the implication of ROS in energy metabolism could be dependent on the size and concentration of AgNPs and probably cell-line-dependent. In the study by Lee et al., treatment with 5 nm AgNPs increased the ROS production, and, as a result, the lactate release decreased in the HepG2 and Huh7 cell lines [56]. However, Chen et al. showed how 25 nm AgNPs induced an increase in lactate production without implication of ROS production in HEK293T [52].

Additionally, to direct the effect on ROS production by NPs, a probable role of NPs in cancer treatment is the possibility of acting as carriers of molecules, which modulates oxidative state. Ever since the study by Jang et al. showed that resveratrol (RSV) can act as an antioxidant and antimutagen compound and can restrain oncogenesis in animal models [57], there have been many studies about these properties. An antitumor effect has been related to several signaling pathways, including inhibiting cancer cell glucose metabolism, by a reduction in reactive oxygen species (ROS)-mediated hypoxia inducible factor-1a activation [58]. However, its application for in vivo cancer treatment is restricted, due to its water insolubility and fast metabolism. Therefore, due to its lipophilicity, various formulations, such as PEG-poly(ε-caprolactone) copolymer (PEG-PCL)-based NPs [59], PEG-Polylactide-block (PEG-PLA) NPs [58,60], and lipid NPs, including solid lipid nanoparticles (SLNs), liposomes, and nanostructured lipid carriers (NLCs) [61,62], have been tested to improve their in vivo delivery and stability. Treatment of colon cancer cells with RSV-loaded PEG-PLA polymeric NPs decrease survival, increasing apoptosis and reducing glucose uptake, mediated by the reduction in the intracellular ROS concentration [58] (Figure 1).

These studies have shown relevant results to use NPs as a cancer therapy, most of them through the increase in ROS; however, the increase in ROS as a cancer therapy is controversial because some ROS levels have been reported to promote cancer growth; additionally, cancer cells can counteract some levels of ROS by generating reduced glutathione (GSH) [63]. Considering these facts, using the increase in ROS as a cancer therapy should exceed the threshold that induces detrimental effects on the cells and cannot be countered by antioxidant defenses. A study by Kong Ong et al. describes a nanosystem that can make it possible. They describe the synthesis of an organically modified silica nanosystem (ORMOSIL@GOx) in two critical components, a bornate ester-protected quinone methide silane ligand cocondensed with tetraethyl orthosilicate (TEOS) in a templated sol–gel methodology and a postmodification with (3-aminopropyl)triethoxysilane-conjugated glucose oxidase (APTES-GOx). It is broadly known that cancer cells show increased levels of hydrogen peroxide, which make it possible to cleave the bornate ester-protected quinone methide silane to release p-quinomethane (QM), which reacts with GSH to form a GSH-QM adduct, depleting the level of GSH. To increase the level of hydrogen peroxide, the complex includes a glucose oxidase (GOx), an enzyme able to catalyze intracellular glucose in cancer cells to generate hydrogen peroxide. Additionally, for the generation of ROS from hydrogen peroxide, this reaction also reduces the glucose levels and consequently starves cancer cells. This suppression of antioxidants (GSH) and increase in ROS, increases the oxidative stress level in cancer cells, surpassing the apoptosis threshold [64].

##### Undesired Effects on the Carbohydrate Metabolism in Cancer Studies due to NPs

One of the limitations of these NPs and nanosystems is the specific focus on certain tumor cells and the lack of toxicological evaluation on nontumor cells. Side effects in nontargeted cells or organs should be considered and studied, and specific features of NPs should be exploited to reduce them. As we have previously explained, NPs can be conjugated with targeting ligands to target cells and their microenvironment, with high specificity and affinity [29,30,35,42].

An example of a promising antitumor NP therapy inducer of detrimental effects in other organs is cobalt oxide (Co_3_O_4_). Co_3_O_4_ NPs have been postulated as new opportunities for anticancer drug development against T-cell lymphoma and oral carcinoma, facilitating the apoptosis of cancer cell (Jurkat and KB cells, respectively) [65]. However, the effect of sublethal doses of Co_3_O_4_ NPs on the brain of mice treated via enteral for 30 days has been evaluated showing also alterations of the pentose phosphate pathway (PPP). Co_3_O_4_ NPs are retained in the brain promoting a remarkable increase in glucose, pyruvate, lactate, and glycogen levels together with an upregulation of hexokinase, glucose 6 phosphatase, and lactate dehydrogenase activity. However, a reduction in the activity of the glucose 6 phosphate dehydrogenase was detected in the brain showing signs of impairment of the pentose phosphate metabolism. Hence, a reduction in glucose 6 phosphate dehydrogenase activity in the brain relates to an impairment of the pentose phosphate pathway together with a reduction in Nicotinamide Adenine Dinucleotide Phosphate Hydrogen (NADPH) and the defenses against oxidative stress. Co_3_O_4_ NPs induce neural stress and disturbance in the treated animals and increase brain carbohydrate metabolism to meet energy demand [66] (Figure 1).

#### 2.1.2. NP Reprogramming of Carbohydrate Metabolism in Diabetes

##### Beneficial Applications of NPs as Antidiabetic Drugs

As we have previously described, AgNPs are good candidates for anticancer therapy, and they have been described as antidiabetic NPs. AgNPs induce a reduction in glycemia, increase insulin levels and expression, increase glucokinase (GK) activity and expression, and upregulate the insulin receptor-A (IRA) and glucose transporter-2 (GLUT-2) in diabetic rats [67] (Figure 1).

Another promising material to treat diabetes is magnesium. Magnesium is one of the four most abundant cations in living organisms, and it has important roles in reactions catalyzed by more than 300 enzymes, including some related to glucose oxidation, glucose transport, insulin release, and lipid metabolism. In addition, magnesium acts as a catalyzer of the ATPase and adenylate cyclase enzymes. The administration of nanosized magnesium oxide in diabetic mice decreased the concentration of glucose [68].

Additional promising NPs are zinc oxide (ZnO) NPs, which are broadly employed in food additives. Doses of ZnO NPs lower than 10 mg/kg in rats and 8–14 mg/kg in mice, administrated to type 1 and 2 diabetic animals trigger mighty antidiabetic activity (in relation to glucose and insulin levels and glucose tolerance) and antioxidant effects (mediated through enhanced superoxide dismutase (SOD) and catalase activities). Improved glucose tolerance could be a result of several possible mechanisms: (1) a downregulation of the intestinal alpha-glucosidase enzyme and consequently a decrease in glucose absorption, (2) reduction in glycemia as a consequence of the increase in glucose uptake in the liver and its subsequent storage (glycogenesis), and (3) improved glucose disposal [67,69,70,71] (Figure 1).

In addition to NPs that by themselves can act as antidiabetic drugs, NPs can also be used as carriers, loaded with antidiabetic compounds. Lipid nanoparticles have been tested to deliver small interfering RNA (siRNA) of glucagon receptors in diabetic mouse models, showing promising results in the improvement of glucose homeostasis [72]. Chitosan–whey protein nanoparticles loaded with the tamarind trypsin inhibitor have been able to reduce fasting glycemia without compromising insulinemia [73].

##### Undesired Effects of NPs Used in Food Industry on Carbohydrate Metabolism in Relation to Glycemia and Insulin Resistance

Although NP treatments can be promising therapies as antidiabetic drugs, NPs used in the food industry can induce an increase in glycemia and insulin resistance. Titanium dioxide (TiO_2_) NP (E171) is a permitted additive for coloring foods, sweets, or candies and is employed in pharmaceutical and personal care products [74]; however, their safety should be reconsidered. The oral intake of 24 nm spherical and anatase TiO_2_ NPs (doses based for children up to the age of 10 in the US) in rats could induce liver, kidney, and heart damage as well as alterations in the white and red blood cell counts. TiO_2_ NPs in combination with high doses of glucose (mimicking sugar intake among U.S. children and adolescents) facilitate synergistic toxicological effects. The synergistic toxicity could be due to the adsorption of glucose onto the surface of TiO_2_ NPs, resulting in an increased cellular uptake of glucose [75]. The toxic effects of NPs can also be the consequence of ROS alterations. TiO_2_ NPs induce endoplasmic reticulum stress (ER stress) and disturb the mono-oxygenase system, increasing ROS levels [76]. Afterward, ROS activates inflammatory cytokines and phosphokinases, resulting in the phosphorylation of the insulin receptor substrate 1 and, consequently, insulin resistance, resulting in an increase in plasma glucose. It is important to remark that TiO_2_ NPs increased glucose in plasma, and this was not reverted after discontinuation of oral administration of TiO_2_ NPs. The persistently high glucose concentration increased ROS levels, and ROS maintain the insulin resistance and consequently high glycemia. However, intake of TiO_2_ NPs was not able to induce b-cell apoptosis in the pancreas, denoting that the glycemia increase was due to insulin resistance [77] (Figure 1).

TiO_2_ NPs are not the only metal oxide nanoparticles that can increase ROS levels and affect the glucose homeostasis. Silicon dioxide (SiO_2_) NPs are another example of NPs used in the food industry, although their use is in food packaging, flavor carriers, and adsorbent or clarifying agents [78], and they have been widely applied as vehicles for drug delivery [79]. Although SiO NPs have not been used as an ingredient of food products, previous studies have shown the possibility of nanomaterial migration from packaging or containers to foodstuff [78]. A recent study has described similar alterations on the metabolism from SiO_2_ NPs, after 10 weeks of 100 mg/kg via oral administration in mice, with a significant increase in blood glucose levels. The increase in glucose is a consequence of the same mechanism as the increase by TiO_2_ NP- and SiO_2_ NP-induced insulin resistance through ER stress and the generation of ROS [80,81] (Figure 1). The generation of ROS and cellular damage in the vascular system has been shown by administrating low doses of SiO_2_ NPs (7–35 mg/kg) [82].

Another example is zinc oxide (ZnO) NPs, which are also widely used in food additives. In this case, the toxicity or beneficial effects seems to be related to the doses. Although doses of ZnO NPs lower than 10 mg/kg in rats and 8–14 mg/kg in mice show beneficial effects in terms of glycemia, as we have previously described [67,69,70,71], doses of ZnO NPs equal to or above 25 mg/kg induce ER stress and increased ROS and therefore elevate plasma glucose in mice [83]. (Figure 1).

### 2.2. Implications in Lipid Metabolism

**Figure 2:**
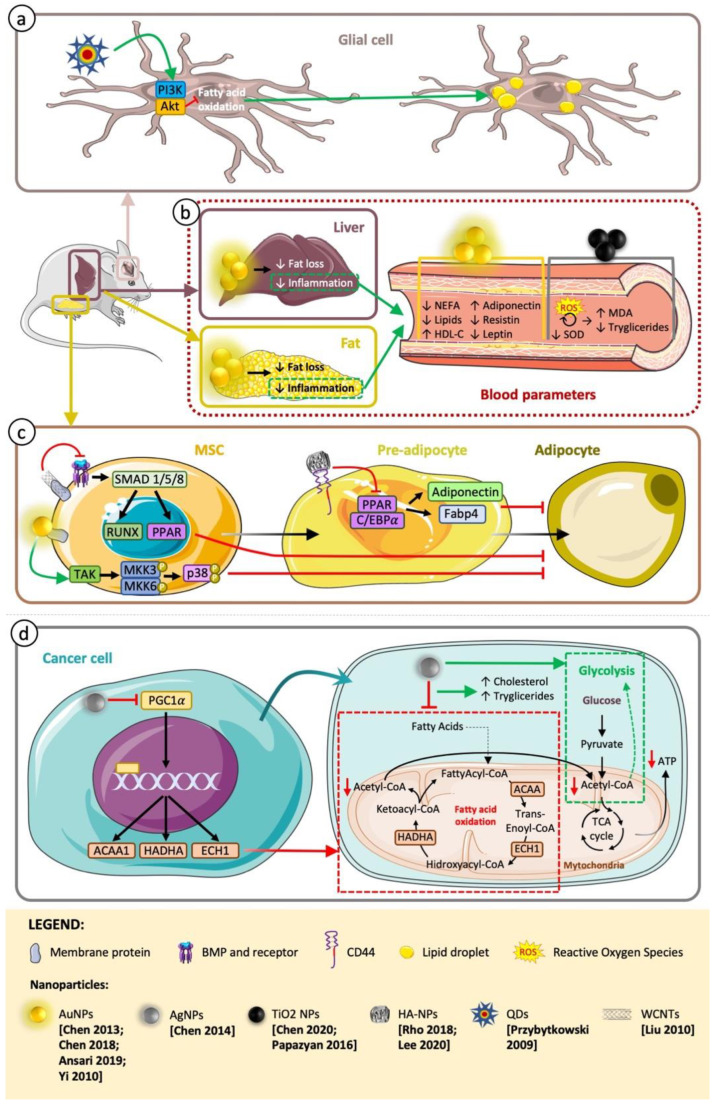
Effect of NPs in lipid metabolism. Role of NPs in different tissues (brain, liver, fat, and MSCs). Red lines are negative stimuli (decrease in metabolite or enzyme activity), and green lines are positive stimuli (increase in metabolite or enzyme activity). (**a**) QDs activate the PI3K/Akt pathway, blocking fatty acid oxidation, resulting in accumulation of lipid droplets in glial cells. (**b**) AuNPs reduce fat and inflammation in liver and fat, tissue improving blood parameters. On the other hand, AgNPs produce ROS and reduce SOD, leading to MDA accumulation and reduction in blood triglycerides. (**c**) AuNPs, WCNTs, and HA-NPs by modulation of TAK, BMP, and PPAR pathways, respectively, reduce the differentiation of MSCs to adipocytes. (**d**) In eukaryotic cells, AgNPs inhibit PGC1a reducing the fatty acid oxidation and redirecting the energy production to glycolysis. Figure was partly generated using Servier Medical Art, provided by Servier, licensed under a Creative Commons Attribution 3.0 unported license [52,87,88,89,90,92,93,97,98,100,103,104].

Lipids have three major roles: energy storage, cell membrane structure, and cell signaling [47]. Triglycerides and cholesterol are the major lipids circulating in the blood. Cholesterol guarantees the stability of cell membranes and the generation of indispensable molecules, such as hormones and vitamin D. Triglycerides are necessary to maintain the energy balance. With the aim of obtaining energy from lipids, triglycerides are metabolized by hydrolysis, generating fatty acids and glycerol. Lipid metabolism is associated with carbohydrate metabolism, as glycerol enters in glycolysis as Dihidroxiacetona-Posphate (DHA-P), and the fatty acid metabolism (fatty acid oxidation or beta (β)-oxidation) produces the acetyl coenzyme A (Acetyl CoA), which enters the Krebs cycle and is employed to produce ATP, as acetyl CoA derived from pyruvate. Additionally, products from glucose (such as acetyl CoA) can be transformed into lipids. The energy obtained from triglycerides duplicates the energy per unit mass than that obtained from carbohydrates and proteins. As consequence, when glucose levels decrease, triglycerides are transformed in acetyl CoA with the aim of generating ATP through aerobic respiration [84]. Additionally, for modifications on carbohydrate metabolism, NPs can also induce lipid modifications (Figure 2).

#### 2.2.1. Therapeutic Use of NPs as Antiobesity Agents

Carbohydrate metabolism reprogramming can be used to attack cancer cells, and lipid reprogramming can be a therapeutic approach against obesity. Therapies based on nanotechnology have been studied as another option to defeat obesity, avoiding the secondary side effects related to traditional therapies [85].

##### Desired Antiobesity NP Effect by Inflammation Modulation

Gold nanoparticles (AuNPs) are remarkable components for applications in biomedicine. AuNPs have been broadly used for diagnostics, and their use as therapeutic NPs has been increasing [86] thanks to their biocompatibility, low cytotoxicity, and cell regulatory effects, including modulation of lipid metabolism [87,88,89,90]. It has been shown how 21 nm spherical AuNPs intraperitoneally accumulate within the abdominal fat tissue and liver and cause a significant reduction in fat and a downregulation of inflammatory signals (reduction in tumor necrosis factor-α (TNFα) and interleukin-6 (IL-6) mRNA levels) without measurable organ or cell toxicity in the liver and kidneys in healthy C57BL/6 male mice [87]. However, this beneficial effect is not just restricted to healthy mice. AuNPs reduce hyperlipidemia and improve glucose tolerance in mice fed a high-fat diet (HFD) [88] and in obese rodents [89,90]. After treatment with a high-fat diet with AuNPs, the body weight and retroperitoneal fat mass of mice are reduced, plasma nonesterified fatty acid (NEFA) levels are reduced, high-density lipoprotein cholesterol (HDL-C) levels are significantly elevated, and plasma lipid metabolic markers are improved. Proinflammatory markers do not show any improvement (reduction) in fat after AuNP treatment; however, they are reduced in the liver (TNFα and toll-like receptor 4 (TLR-4)), and lipid metabolic markers are improved in fat [88]. Obese rats treated with 21 nm AuNPs ameliorate the lipid profile, atherogenic and coronary indexes, liver markers, inflammatory markers, hormones, such as leptin, resistin, and adiponectin, and restore the internal membrane, nuclei, and damaged cytoplasm [90]. The underlying mechanism may be attributed to a reduction in proinflammatory cytokines in fat and liver [88,89,90]. The impaired generation of proinflammatory molecules (so-called “adipokines”) by adipose tissue has been related to the metabolic side effects of obesity [91]; therefore, AuNPs offer a new opportunity as a therapeutic agent for obesity by targeting inflammation (Figure 2).

##### Antiobesity NP Effect by Adipocyte Differentiation and Maturation Modulation

Hyaluronic acid nanoparticles (HA-NPs) have been also postulated as potential therapeutic agents for obesity and related metabolic anomalies (Figure 2). CD44 is a multifunctional membrane receptor and the major cell surface HA receptor, meaning that HA-based NPs are able to directionally attack cells that overexpress CD44 [92,93]. Latterly, it has been shown that CD44 is overexpressed in adipose tissue by HFD and that the effective targeting by HA-NPs of adipose tissues is mediated by the interaction between HA-NPs and CD44. 3T3-L1 preadipocytes treated with HA-NPs result in a dose-dependent suppression of adipocyte differentiation and a maturation decrease proliferator-activated receptor gamma (PPARγ) expression, CCAAT/enhancer-binding protein alpha (C/EBPα), fatty acid-binding protein 4 (FABP4), and adiponectin (AdipoQ) and also reduce the lipid accumulation by downregulating fatty acid synthase (FAS) and stearoyl-CoA desaturase-1 (SCD1). Treatment of diet-induced obese mice with HA-NPs (150 mg/kg) daily for 30 days, in addition to these previously named effects, reduces body weight and epididymal fat mass [93].

#### 2.2.2. Toxicity of NPs in Relation to Lipid Metabolism and Adipocyte Differentiation

##### Undesired Effects of Therapeutic NPs

NPs have been associated with the differentiation of mesenchymal stem cells (MSCs), which can differentiate to several cell lineages under specific in vitro conditions: ectodermal, mesodermal (such as adipocytes, osteocytes, and chondrocytes), and endodermal [94,95,96]; however, the disruption of MSC differentiation leads to multiple degenerative diseases [96].

As we have previously described, AuNPs offer a new opportunity as a potential drug to treat obesity [87,88,89,90]; however, understanding the interplay between nanomaterials and cells is necessary for managing these interactions for applications in biomedicine, preventing undesirable effects. AuNPs induce the differentiation of MSCs toward osteoblast cells over adipocyte cells. AuNPs interact with the cell membrane and bind to cytoplasmic proteins, inducing mechanical stress and the activation of p38 mitogen-activated protein kinase pathway (MAPK) signaling pathway, inducing an improved osteogenic transcriptional profile and a downregulated adipogenic transcriptional profile [97] (Figure 2).

Another example of NPs able to downregulate the adipogenic differentiation of mesenchymal stem cells is the carboxylated single-walled carbon nanotubes (SWCNTs) and the carboxylated multiwalled carbon nanotubes (MWCNTs). They can interact with cell membrane and cytoplasmic proteins and through a Smad-dependent bone morphogenetic protein (BMP) signaling pathway inhibiting adipogenic differentiation of MSCs [98] (Figure 2).

Adipogenesis is maintained during the whole life of adipose tissue, with continual differentiation of preadipocytes, indispensable cells to preserve tissue functions during aging, and the alteration of MSC differentiation by NPs could lead to deleterious effects on adipose aging [99].

##### Undesired Effects of Diagnostic NPs

Beyond the use of NPs for therapy, NPs can be used as tools for medical imaging. In this context, quantum dots (QDs), colloidal semiconductor NPs with exceptional luminescence features, are very attractive. Nevertheless, some QDs can induce damage to the cells, particularly if their surface is not fully protected or if they degrade within the biological environment. The toxicity and implication in lipid metabolism of poorly fluorescent cadmium telluride (CdTe) NPs, without zinc sulfide (ZnS) capping but with cysteamine coating, as well as highly fluorescent CdSe/ZnS NPs, capped with ZnS and coated with cysteamine on the surface, were analyzed using a primary mouse hypothalamic culture of glial cells. An analysis of lipids revealed the accumulation of lipids in cytoplasmic lipid droplets (LDs) by de novo lipid synthesis, which was dependent on signaling via phosphatidylinositol 3-kinase (PI3K)/erine/threonine protein kinase (AKT) and trophic factors. The effect on LD accumulation was dose-dependent and much higher with uncoated CdTe QDs. The reduction in the oxidation of fatty acids induced by NPs can explain the formation of LDs, which happens simultaneously with an increase in LDs. It is worth stressing that alterations in the intracellular lipid metabolism occur without jeopardizing cellular viability. However, it is not an insignificant alteration. Excess levels of cytoplasmic LDs in nonadipose cells are considered harmful and may be related to several human pathologies, such as fatty liver, obesity, atherosclerosis, and type 2 diabetes, and may take part in the progression of insulin resistance and lipotoxic tissue damage [100] (Figure 2).

##### Undesired Effects of NPs Used in Food and Personal Care Products

In addition to the use of NPs for therapy and diagnosis, NPs are also used, as we have previously described, as additives. Titanium dioxide (TiO_2_) NP (E171) is an authorized additive used in foods and pharmaceutical and personal care products [74]. It has been described as an antimicrobial compound due to the bacterial killing by oxidative stress because of the generation of reactive oxygen species [101,102]. This effect is not limited to prokaryotes; in eukaryotic cells, it is able to induce oxidative stress and consequently alter the lipid metabolism [103]. Daily ingestion of 29 nm of spherical and anatase crystals titanium dioxide (TiO_2_) NPs for 90 days by Sprague–Dawley rats altered the lipid metabolism in a dose-dependent manner and derived an oxidative stress induction. The alterations of the lipid metabolism include a reduction in triglycerides in blood and significant changes in the lipidomic signature in the serum, including a significant alteration of the glycerophospholipid metabolism pathway. TiO_2_ NPs also induced the accumulation of the lipid peroxidation product (MDA) and downregulated the antioxidant enzyme SOD, indicating that the redox balance was disrupted [103]. Previous studies revealed the accumulation of lipid intermediates, and low levels of triglycerides generated oxidative stress, inflammation, and cell damage [104] that could produce a positive feedback and a maintenance of lipid metabolism alterations (Figure 2).

AgNPs are well known for their interesting antimicrobial properties and are widely used in different areas. However, there are growing concerns on the side effects for the environment and human health. A study by Chen et al. explains how AgNPs coated with polyvinylpyrrolidone under sublethal concentrations might incur an adaptive shunt of the energy metabolism mode to glycolysis, accompanied by a significant decrease in the lipid metabolism. AgNPs disrupt several metabolism-related genes, among them, PGC-1a, suppressing fatty acid oxidation, inhibiting the metabolism of fatty acids to acetyl-coA, and consequently reducing the initial substrate of the Krebs cycle. PGC-1a induces the upregulation of genes related to gluconeogenesis and fatty acid oxidation, drives cholesterol metabolism through the activation of LXRa (liver X receptor alpha), and improves mitochondrial biogenesis and activation of Cyp7A1 (cholesterol 7alpha-hydroxylase) expression. An analysis of the targeted genes by PGC-1a reveals a significant decline in mRNA levels on the genes involved in fatty acid oxidation in a dose-dependent manner, including ACAA1 (acetyl-CoA acyltransferase 1), ACAA2 (acetyl-CoA acyltransferase 2), HADHA (hydroxyacyl-CoA dehydrogenase), and ECH1 (enoyl-coenzyme A hydratase 1). Therefore, AgNP-treated HepG2, HeLa, A498, PC3, and HEK293T cells compared to untreated cells significantly decrease ATP production associated with an increase in intracellular triglyceride and cholesterol accumulations in a dose-dependent manner. The lack of ATP production could oblige the cells to reprogram energy metabolism, increasing glycolysis. Different AgNP sizes (20 to 45 nm) and shapes (spherical and plate-like) have shown similar alterations on the cellular energy metabolism, as manifested by triglyceride and cholesterol accumulations; however, smaller AgNPs induced a stronger effect, probably due to being more prone to enter cells [52] (Figure 2).

## 3. Conclusions

NPs have unique intrinsic properties that make them useful tools for a wide range of applications in biomedicine, including diagnosis, treatment, and theragnosis [2]. Additionally, recent progress has been made in the field of drug delivery systems, developing nanocarriers capable to release their cargo in a site- and time-specific way, showing interesting advantages for the novel treatments. These nanocarriers need to be sensitive to external (light, ultrasound, and magnetic field) or internal (pH, redox, and enzyme) stimuli, for their cargo release [105]. Therefore, even though NPs have been used in the clinical setting since the early1990s, the tremendous potential for developing novel disease-specific treatments with reduced side effects (due to their controlled release) has to be accompanied with increasing concerns about their biosafety; this has reduced the number of NPs approved for diagnosis to only one type, namely iron oxide NPs [2,3,21,22,23,24,25,26,27].

Focusing on cancer, tumor cells have special features; specifically, metabolic abnormalities are a hallmark of cancer. One of the metabolic features of cancer cells is the increased glucose uptake and consumption via aerobic glycolysis, which can be exploited to specifically target cancer cells. The use of NPs can be directly directed to reduce glucose uptake, decreasing ATP generation. Additionally, to direct metabolic carbohydrate reprogramming, NPs can induce the generation of ROS modifying the glucose metabolic pathways, indirectly reducing the energy production. The alteration of lipid metabolism is another way to target cancer cells; lipid metabolism produces metabolites implicated in glycolysis and the Krebs cycle to produce ATP. Therefore, alteration of the lipid metabolism could indirectly reduce the ATP production. NPs can disrupt lipid metabolism and consequently attack cancer cells.

Nevertheless, more extensive studies are needed; many of these NPs were designed to treat or diagnose diseases, especially cancer, or have been used as additives in food or the self-care industry, but the possibility of undesirable secondary effects should be considered. In vivo experiments would be necessary to test the “real” effect of the nanoparticles in the whole organism avoiding missing relevant information about the biosecurity of the tested NPs. A clear example of how NPs can disrupt several metabolic pathways is also derived from the production of ROS. Several NPs have been related to ROS production, which can increase glycemia, which can ultimately promote oncogenesis, tumor resistance, and diabetes.

Initial studies used to focus on a selected target; nevertheless, more extensive studies are needed to understand their role in the metabolism of other compounds, avoiding missing relevant information about the biosecurity of the tested NPs.

The preclinical development of novel nanotechnology-formulated drugs is challenging, mainly due to the need for a deep physicochemical characterization of the compounds for higher safety and efficacy. With more information coming from those studies, the design and development of other pharmaceutical products might be different.

Nevertheless, NP translation into the clinical setting is particularly complex, because of (l) the biochemically sophistication of the strategies and (ll) the lack of worldwide-accepted protocols to test the NPs before the initiation of a clinical trial. The continuous production of new materials lacking in safety information, makes it difficult to harmonize procedures or regulatory guides to perform preclinical tests [106,107]. Consequently, there is a need for the development of accepted and specific guidelines including the identification of the possible metabolic alterations of NPs (related to their size, composition, etc.) and their interaction with the immune system, independent of their application.

Other factors to take into consideration for new, NP-based therapies in clinical trials could be the inclusion of genomic heterogeneity, commensal diversity, sexual dimorphism, and biological aging on the studies, which have been largely ignored in traditional nanomedicine experiments [108].

This would increase our knowledge, leading to safer use and reduced toxicity of NPs, thereby increasing the number of available NPs for diagnosis, treatment, and theragnosis of human diseases.

## Figures and Tables

**Table 1:** Nanostructured material classification and description of the categories.

Features	Categories	Description
Origin [4,5,6,7,8,9,10]	Natural	Erosion and dust storms, volcanic activity, forest fires, and from biogenic sources, e.g. shed skin and hair and o bioreductively formed deposits of elements in certain bacteria.
	Synthetic	Pollutant: Simple combustion, food cooking, industrial manufacturing, combustion (in vehicle and airplane engines and for power generation).
		Intentionally produced: pesticides and fertilizers, cosmetic and personal care products, tires, clothing, water-repellent products, food additives and treatment and diagnosis in medicine.
Dimensionality (Electron movement) [1,11,12,13]	0D	Quantum dots, nanoparticles arrays, core-shell nanoparticles, hollow cubes and nanospheres.
	1D	Nanowires, nanorods, nanotubes, nanobelts, nanoribbons, and hierarchical nanostructures.
	2D	Junctions, branched structures, nanoprisms, nanoplates, nanosheets, nanowalls and nanodisks.
	3D	Nanoballs(dendritic structures), nanocoils, nanocones, nanopillers and nanoflowers.
Morphology [1,4,14,15]	Flatness, sphericity and aspect ratio	Implications: Internalization rate of a particle and toxicity.
		High-aspect ratio: nanotubes and nanowires.
		Low-aspect ratio: spherical, oval, cubic, prism, helical or pillar morphologies.
Composition (Single material or composite) [4,7,8,9,16,17,18]	Material-based categories	Carbon-based nanomaterials.
		Inorganic-based nanomaterials(metal and metal oxide).
		Organic-based nanomaterials
		Composite-based nanomaterials.
Agglomeration state/Uniformity [4,19,20]	Disperses or agglomerated	The surface properties primarily determine the agglomeration state and uniformity of the particles and therefore their effective size, especially under physiological conditions.
		Implications: cellular uptake, in vivo biodistribution, effective area of metal release and generation of reactive oxygen species generation(ROS),shape into a fractal and toxicity.

## Data Availability

No new data were created.

## References

[1] Jeevanandam J., Barhoum A., Chan Y.S., Dufresne A., Danquah M.K. (2018). Review on nanoparticles and nanostructured materials: History, sources, toxicity and regulations. Beilstein J. Nanotechnol..

[2] Chen N., Wang H., Huang Q., Li J., Yan J., He D., Fan C., Song H. (2014). Long-Term Effects of Nanoparticles on Nutrition and Metabolism. Small.

[3] Li J., Chang X., Chen X., Gu Z., Zhao F., Chai Z., Zhao Y. (2014). Toxicity of inorganic nanomaterials in biomedical imaging. Biotechnol. Adv..

[4] Buzea C., Pacheco I.I., Robbie K. (2007). Nanomaterials and nanoparticles: Sources and toxicity. Biointerphases.

[5] Buzea C., Pacheco I., Ghorbanpour M., Manika K., Varma A. (2017). Nanomaterial and Nanoparticle: Origin and Activity. Nanoscience and Plant-Soil Systems. Soil Biology.

[6] Griffin S., Masood M.I., Nasim M.J., Sarfraz M., Ebokaiwe A.P., Schäfer K.-H., Keck C.M., Jacob C. (2017). Natural Nanoparticles: A Particular Matter Inspired by Nature. Antioxidants.

[7] Lee J.E., Lee N., Kim T., Kim J., Hyeon T. (2011). Multifunctional Mesoporous Silica Nanocomposite Nanoparticles for Theranostic Applications. Accounts Chem. Res..

[8] Jana N.R., Ray S.C. (2015). Chapter 3—Graphene-Based Carbon Nanoparticles for Bioimaging Applications. Applications of Graphene and Graphene-Oxide Based Nanomaterials Micro and Nano Technologies.

[9] Nazıroğlu M., Muhamad S., Pecze L. (2017). Nanoparticles as potential clinical therapeutic agents in Alzheimer’s disease: Focus on selenium nanoparticles. Expert Rev. Clin. Pharmacol..

[10] Shahbazi M.-A., Faghfouri L., Ferreira M.P.A., Figueiredo P., Maleki H., Sefat F., Hirvonen J., Santos H.A. (2020). The versatile biomedical applications of bismuth-based nanoparticles and composites: Therapeutic, diagnostic, biosensing, and regenerative properties. Chem. Soc. Rev..

[11] Gleiter H. (1995). Nanostructured Materials: State of the Art and Perspectives. Nanostructured Materials.

[12] Pokropivny V.V., Skorokhod V.V. (2007). Classification of nanostructures by dimensionality and concept of surface forms engineering in nanomaterial science. Mater. Sci. Eng. C.

[13] Tiwari J.N., Tiwari R.N., Kim K.S. (2012). Zero-dimensional, one-dimensional, two-dimensional and three-dimensional nanostructured materials for advanced electrochemical energy devices. Prog. Mater. Sci..

[14] Ramos A.P., Cruz M.A.E., Tovani C.B., Ciancaglini P. (2017). Biomedical applications of nanotechnology. Biophys. Rev..

[15] Pacheco-Blandino I., Vanner R., Buzea C. (2012). Toxicity of nanoparticles. Toxic. Build. Mater..

[16] Ray S.C., Jana N.R. (2017). Chapter 5—Application of Carbon-Based Nanomaterials as Drug and Gene Delivery Carrier. Carbon Nanomaterials for Biological and Medical Applications Micro and Nano Technologies.

[17] Kumar R., Lal S. (2014). Synthesis of Organic Nanoparticles and their Applications in Drug Delivery and Food Nanotechnology: A Review. J. Nanomater. Mol. Nanotechnol..

[18] Wang Y., Huang L. (2014). Composite nanoparticles for gene delivery. Adv. Genet..

[19] Bantz C., Koshkina O., Lang T., Galla H.-J., Kirkpatrick C.J., Stauber R.H., Maskos M. (2014). The surface properties of nanoparticles determine the agglomeration state and the size of the particles under physiological conditions. Beilstein J. Nanotechnol..

[20] Zook J.M., MacCuspie R., Locascio L.E., Halter M.D., Elliott J.T. (2010). Stable nanoparticle aggregates/agglomerates of different sizes and the effect of their size on hemolytic cytotoxicity. Nanotoxicology.

[21] Anselmo A.C., Mitragotri S. (2019). Nanoparticles in the clinic: An update. Bioeng. Transl. Med..

[22] Anik M.I., Mahmud N., Al Masud A., Hasan M. (2021). Gold nanoparticles (GNPs) in biomedical and clinical applications: A review. Nano Sel..

[23] Wei H., Hu Y., Wang J., Gao X., Qian X., Tang M. (2021). Superparamagnetic Iron Oxide Nanoparticles: Cytotoxicity, Metabolism, and Cellular Behavior in Biomedicine Applications. Int. J. Nanomed..

[24] Saji V.S. (2022). Supramolecular organic nanotubes for drug delivery. Mater. Today Adv..

[25] Othman A.K., El Kurdi R., Badran A., Mesmar J., Baydoun E., Patra D. (2022). Liposome-based nanocapsules for the controlled release of dietary curcumin: PDDA and silica nanoparticle-coated DMPC liposomes enhance the fluorescence efficiency and anticancer activity of curcumin. RSC Adv..

[26] Cheng C., Qiao J., Zhang H., Zhao Z., Qi L. (2022). Polymer-capped gold nanoparticles as nanozymes with improved catalytic activity for the monitoring of serum ciprofloxacin. Analyst.

[27] Joy R., George J., John F. (2022). Brief Outlook on Polymeric Nanoparticles, Micelles, Niosomes, Hydrogels and Liposomes: Preparative Methods and Action. Chem. Sel..

[28] Baetke S.C., Lammers T., Kiessling F. (2015). Applications of nanoparticles for diagnosis and therapy of cancer. Br. J. Radiol..

[29] Petros R.A., DeSimone J.M. (2010). Strategies in the design of nanoparticles for therapeutic applications. Nat. Rev. Drug Discov..

[30] Banerjee A., Qi J., Gogoi R., Wong J., Mitragotri S. (2016). Role of nanoparticle size, shape and surface chemistry in oral drug delivery. J. Control. Release.

[31] Chen L., Xiao S., Zhu H., Wang L., Liang H. (2016). Shape-dependent internalization kinetics of nanoparticles by membranes. Soft Matter.

[32] Zhang B., Feng X., Yin H., Ge Z., Wang Y., Chu Z., Raabova H., Vavra J., Cigler P., Liu R. (2017). Anchored but not internalized: Shape dependent endocytosis of nanodiamond. Sci. Rep..

[33] Wang W., Gaus K., Tilley R.D., Gooding J.J. (2019). The impact of nanoparticle shape on cellular internalisation and transport: What do the different analysis methods tell us?. Mater. Horizons.

[34] Bhattacharya S., Anjum M., Patel K.K. (2022). Gemcitabine cationic polymeric nanoparticles against ovarian cancer: Formulation, characterization, and targeted drug delivery. Drug Deliv..

[35] Nie S. (2010). Understanding and overcoming major barriers in cancer nanomedicine. Nanomedicine.

[36] Kamaly N., Farokhzad O.C., Corbo C. (2022). Nanoparticle protein corona evolution: From biological impact to biomarker discovery. Nanoscale.

[37] Suk J.S., Xu Q., Kim N., Hanes J., Ensign L.M. (2016). PEGylation as a strategy for improving nanoparticle-based drug and gene delivery. Adv. Drug Deliv. Rev..

[38] Shi D., Beasock D., Fessler A., Szebeni J., Ljubimova J.Y., Afonin K.A., Dobrovolskaia M.A. (2021). To PEGylate or not to PEGylate: Immunological properties of nanomedicine’s most popular component, polyethylene glycol and its alternatives. Adv. Drug Deliv. Rev..

[39] Betker J.L., Anchordoquy T.J. (2020). The Use of Lactose as an Alternative Coating for Nanoparticles. J. Pharm. Sci..

[40] Yang B., Jiang J., Jiang L., Zheng P., Wang F., Zhou Y., Chen Z., Li M., Lian M., Tang S. (2020). Chitosan mediated solid lipid nanoparticles for enhanced liver delivery of zedoary turmeric oil in vivo. Int. J. Biol. Macromol..

[41] Wang W., Meng Q., Li Q., Liu J., Zhou M., Jin Z., Zhao K. (2020). Chitosan Derivatives and Their Application in Biomedicine. Int. J. Mol. Sci..

[42] Gao H., Yang Z., Zhang S., Cao S., Shen S., Pang Z., Jiang X. (2013). Ligand modified nanoparticles increases cell uptake, alters endocytosis and elevates glioma distribution and internalization. Sci. Rep..

[43] Groneberg D., Giersig M., Welte T., Pison U. (2006). Nanoparticle-Based Diagnosis and Therapy. Curr. Drug Targets.

[44] Encabo-Berzosa M.M., Sancho-Albero M., Sebastian V., Irusta S., Arruebo M., Santamaria J., Martin Duque P. (2017). Polymer functionalized gold nanoparticles as nonviral gene delivery reagents. J. Gene Med..

[45] Agasti S.S., Rana S., Park M.-H., Kim C.K., You C.-C., Rotello V.M. (2010). Nanoparticles for detection and diagnosis. Adv. Drug Deliv. Rev..

[46] Brun E., Roselli C.S. (2014). Could nanoparticle corona characterization help for biological consequence prediction?. Cancer Nanotechnol..

[47] Cooper G.M., NCBI (2000). The Cell: A Molecular Approach.

[48] Kaelin W., Thompson C. (2010). Clues from cell metabolism. Nature.

[49] Nature. Cell Origins and Metabolism. Editor(s): Gary Coté, Mario De Tullio. https://www.nature.com/scitable/topic/cell-origins-and-metabolism-14122694/.

[50] Warburg O. (1956). On the origin of cancer cells. Science.

[51] Potter M., Newport E., Morten K.J. (2016). The Warburg effect: 80 years on. Biochem. Soc. Trans..

[52] Chen Y., Wang Z., Xu M., Wang X., Liu R., Liu Q., Zhang Z., Xia T., Zhao J., Jiang G. (2014). Nanosilver Incurs an Adaptive Shunt of Energy Metabolism Mode to Glycolysis in Tumor and Nontumor Cells. ACS Nano.

[53] Jin L., Zhou Y. (2019). Crucial role of the pentose phosphate pathway in malignant tumors (Review). Oncol. Lett..

[54] Xu B., Zhang Q., Luo X., Ning X., Luo J., Guo J., Liu Q., Ling G., Zhou N. (2020). Selenium nanoparticles reduce glucose metabolism and promote apoptosis of glioma cells through reactive oxygen species-dependent manner. Neuroreport.

[55] Raj A., Shah P., Agrawal N. (2017). Sedentary behavior and altered metabolic activity by AgNPs ingestion in Drosophila melanogaster. Sci. Rep..

[56] Lee M.J., Lee S.J., Yun S.J., Jang J.-Y., Kang H., Kim K., Choi I.-H., Park S. (2015). Silver nanoparticles affect glucose metabolism in hepatoma cells through production of reactive oxygen species. Int. J. Nanomed..

[57] Jang M., Cai L., Udeani G.O., Slowing K.V., Thomas C.F., Beecher C.W.W., Fong H.H.S., Farnsworth N.R., Kinghorn A.D., Mehta R.G. (1997). Cancer Chemopreventive Activity of Resveratrol, a Natural Product Derived from Grapes. Science.

[58] Jung K.-H., Lee J.H., Quach C.H.T., Paik J.-Y., Oh H., Park J.W., Lee E.J., Moon S.-H., Lee K.-H. (2013). Resveratrol Suppresses Cancer Cell Glucose Uptake by Targeting Reactive Oxygen Species–Mediated Hypoxia-Inducible Factor-1α Activation. J. Nucl. Med..

[59] Shao J., Li X., Lu X., Jiang C., Hu Y., Li Q., You Y., Fu Z. (2009). Enhanced growth inhibition effect of Resveratrol incorporated into biodegradable nanoparticles against glioma cells is mediated by the induction of intracellular reactive oxygen species levels. Colloids Surf. B Biointerfaces.

[60] Wang J., Li S., Han Y., Guan J., Chung S., Wang C., Li D. (2018). Poly(Ethylene Glycol)–Polylactide Micelles for Cancer Therapy. Front. Pharmacol..

[61] Coimbra M., Isacchi B., van Bloois L., Torano J.S., Ket A., Wu X., Broere F., Metselaar J.M., Rijcken C.J., Storm G. (2011). Improving solubility and chemical stability of natural compounds for medicinal use by incorporation into liposomes. Int. J. Pharm..

[62] Neves A.R., Lúcio M., Martins S., Lima J.L.C. (2013). Novel resveratrol nanodelivery systems based on lipid nanoparticles to enhance its oral bioavailability. Int. J. Nanomed..

[63] Cairns R.A., Harris I.S., Mak T.W. (2011). Regulation of cancer cell metabolism. Nat. Rev. Cancer.

[64] Ong W.K., Jana D., Zhao Y. (2019). A glucose-depleting silica nanosystem for increasing reactive oxygen species and scavenging glutathione in cancer therapy. Chem. Commun..

[65] Chattopadhyay S., Chakraborty S.P., Laha D., Baral R., Pramanik P., Roy S. (2012). Surface-modified cobalt oxide nanoparticles: New opportunities for anti-cancer drug development. Cancer Nanotechnol..

[66] Shaikh S.M., Desai P.V. (2016). Effect of CoO nanoparticles on the carbohydrate metabolism of the brain of mice ‘‘Mus musculus”. J. Basic Appl. Zool..

[67] Alkaladi A., Abdelazim A.M., Afifi M. (2014). Antidiabetic Activity of Zinc Oxide and Silver Nanoparticles on Streptozotocin-Induced Diabetic Rats. Int. J. Mol. Sci..

[68] Naghsh N., Kazemi S. (2014). Effect of Nano-magnesium Oxide on Glucose Concentration and Lipid Profile in Diabetic Laboratory Mice. Iran. J. Pharm. Sci..

[69] Umrani R.D., Paknikar K.M. (2014). Zinc oxide nanoparticles show antidiabetic activity in streptozotocin-induced Type 1 and 2 diabetic rats. Nanomedicine.

[70] El-Gharbawy R.M., Emara A.M., Abu-Risha S.E.-S. (2016). Zinc oxide nanoparticles and a standard antidiabetic drug restore the function and structure of beta cells in Type-2 diabetes. Biomed. Pharmacother..

[71] Siddiqui S.A., Rashid M.O., Uddin G., Robel F.N., Hossain M.S., Haque A. (2020). Jakaria Biological efficacy of zinc oxide nanoparticles against diabetes: A preliminary study conducted in mice. Biosci. Rep..

[72] Neumann U.H., Ho J.S., Chen S., Tam Y.Y.C., Cullis P.R., Kieffer T.J. (2017). Lipid nanoparticle delivery of glucagon receptor siRNA improves glucose homeostasis in mouse models of diabetes. Mol. Metab..

[73] Matias L.L.R., Costa R.O.A., Passos T.S., Queiroz J.L.C., Serquiz A.C., Maciel B.L.L., Santos P.P.A., Camillo C.S., Gonçalves C., Amado I.R. (2019). Tamarind Trypsin Inhibitor in Chitosan–Whey Protein Nanoparticles Reduces Fasting Blood Glucose Levels without Compromising Insulinemia: A Preclinical Study. Nutrients.

[74] Weir A., Westerhoff P., Fabricius L., Hristovski K., von Goetz N. (2012). Titanium Dioxide Nanoparticles in Food and Personal Care Products. Environ. Sci. Technol..

[75] Chen Z., Wang Y., Zhuo L., Chen S., Zhao L., Chen T., Li Y., Zhang W., Gao X., Li P. (2015). Interaction of titanium dioxide nanoparticles with glucose on young rats after oral administration. Nanomedicine.

[76] Hu H., Li L., Guo Q., Zong H., Yan Y., Yin Y., Wang Y., Oh Y., Feng Y., Wu Q. (2018). RNA sequencing analysis shows that titanium dioxide nanoparticles induce endoplasmic reticulum stress, which has a central role in mediating plasma glucose in mice. Nanotoxicology.

[77] Hu H., Li L., Guo Q., Jin S., Zhou Y., Oh Y., Feng Y., Wu Q., Gu N. (2016). A mechanistic study to increase understanding of titanium dioxide nanoparticles-increased plasma glucose in mice. Food Chem. Toxicol..

[78] Kasaai M.R. (2015). Nanosized Particles of Silica and Its Derivatives for Applications in Various Branches of Food and Nutrition Sectors. J. Nanotechnol..

[79] Vallet-Regi M., Colilla M., Izquierdo-Barba I., Manzano M. (2017). Mesoporous Silica Nanoparticles for Drug Delivery: Current Insights. Molecules.

[80] Hu H., Fan X., Guo Q., Wei X., Yang D., Zhang B., Liu J., Wu Q., Oh Y., Feng Y. (2019). Silicon dioxide nanoparticles induce insulin resistance through endoplasmic reticulum stress and generation of reactive oxygen species. Part. Fibre Toxicol..

[81] Shin T.H., Seo C., Lee D.Y., Ji M., Manavalan B., Basith S., Chakkarapani S.K., Kang S.H., Lee G., Paik M.J. (2019). Silica-coated magnetic nanoparticles induce glucose metabolic dysfunction in vitro via the generation of reactive oxygen species. Arch. Toxicol..

[82] Wang D.-P., Wang Z.-J., Zhao R., Lin C.-X., Sun Q.-Y., Yan C.-P., Zhou X., Cao J.-M. (2020). Silica nanomaterials induce organ injuries by Ca2+-ROS-initiated disruption of the endothelial barrier and triggering intravascular coagulation. Part. Fibre Toxicol..

[83] Hu H., Guo Q., Fan X., Wei X., Yang D., Zhang B., Liu J., Wu Q., Oh Y., Feng Y. (2020). Molecular mechanisms underlying zinc oxide nanoparticle induced insulin resistance in mice. Nanotoxicology.

[84] Robin J. (2022). Heyden (advisor). Chapter 24. Metabolism and Nutrition. 164 24.3 Lipid Metabolism. Anatomy and Physiology.

[85] Sibuyi N.R.S., Moabelo K.L., Meyer M., Onani M.O., Dube A., Madiehe A.M. (2019). Nanotechnology advances towards development of targeted-treatment for obesity. J. Nanobiotechnol..

[86] Yeh Y.-C., Creran B., Rotello V.M. (2011). Gold nanoparticles: Preparation, properties, and applications in bionanotechnology. Nanoscale.

[87] Chen H., Dorrigan A., Saad S., Hare D.J., Cortie M.B., Valenzuela S.M. (2013). In Vivo Study of Spherical Gold Nanoparticles: Inflammatory Effects and Distribution in Mice. PLoS ONE.

[88] Chen H., Ng J.P.M., Tan Y., McGrath K., Bishop D.P., Oliver B., Chan Y.L., Cortie M.B., Milthorpe B.K., Valenzuela S.M. (2018). Gold nanoparticles improve metabolic profile of mice fed a high-fat diet. J. Nanobiotechnol..

[89] Chen H., Ng J.P.M., Bishop D.P., Milthorpe B.K., Valenzuela S.M. (2018). Gold nanoparticles as cell regulators: Beneficial effects of gold nanoparticles on the metabolic profile of mice with pre-existing obesity. J. Nanobiotechnol..

[90] Ansari S., Bari A., Ullah R., Mathanmohun M., Veeraraghavan V.P., Sun Z. (2019). Gold nanoparticles synthesized with Smilax glabra rhizome modulates the anti-obesity parameters in high-fat diet and streptozotocin induced obese diabetes rat model. J. Photochem. Photobiol. B Biol..

[91] Weisberg S.P., McCann D., Desai M., Rosenbaum M., Leibel R.L., Ferrante A.W. (2003). Obesity is associated with macrophage accumulation in adipose tissue. J. Clin. Investig..

[92] Rho J.G., Han H.S., Han J.H., Lee H., Nguyen V.Q., Lee W.H., Kwon S., Heo S., Yoon J., Shin H.H. (2018). Self-assembled hyaluronic acid nanoparticles: Implications as a nanomedicine for treatment of type 2 diabetes. J. Control. Release.

[93] Lee W.H., Rho J.G., Han H.S., Kweon S., Nguyen V.Q., Park J.H., Kim W. (2020). Self-assembled hyaluronic acid nanoparticle suppresses fat accumulation via CD44 in diet-induced obese mice. Carbohydr. Polym..

[94] Uccelli A., Moretta L., Pistoia V. (2008). Mesenchymal stem cells in health and disease. Nat. Rev. Immunol..

[95] Pittenger M.F., Discher D.E., Péault B.M., Phinney D.G., Hare J.M., Caplan A.I. (2019). Mesenchymal stem cell perspective: Cell biology to clinical progress. NPJ Regen. Med..

[96] Zhang Q., Dong J., Zhang P., Zhou D., Liu F. (2021). Dynamics of Transcription Factors in Three Early Phases of Osteogenic, Adipogenic, and Chondrogenic Differentiation Determining the Fate of Bone Marrow Mesenchymal Stem Cells in Rats. Front. Cell Dev. Biol..

[97] Yi C., Liu D., Fong C.-C., Zhang J., Yang M. (2010). Gold Nanoparticles Promote Osteogenic Differentiation of Mesenchymal Stem Cells through p38 MAPK Pathway. ACS Nano.

[98] Liu D., Yi C., Zhang D., Zhang J., Yang M. (2010). Inhibition of Proliferation and Differentiation of Mesenchymal Stem Cells by Carboxylated Carbon Nanotubes. ACS Nano.

[99] Dasuri K., Zhang L., Ebenezer P., Fernandez-Kim S.O., Bruce-Keller A.J., Szweda L.I., Keller J.N. (2011). Proteasome alterations during adipose differentiation and aging: Links to impaired adipocyte differentiation and development of oxidative stress. Free Radic. Biol. Med..

[100] Przybytkowski E., Behrendt M., Dubois D., Maysinger D. (2009). Nanoparticles can induce changes in the intracellular metabolism of lipids without compromising cellular viability. FEBS J..

[101] Chen F., Yang X., Wu Q. (2009). Photocatalytic Oxidation of *Escherischia coli*, *Aspergillus niger*, and Formaldehyde under Different Ultraviolet Irradiation Conditions. Environ. Sci. Technol..

[102] Sohm B., Immel F., Bauda P., Pagnout C. (2015). Insight into the primary mode of action of TiO_2_ nanoparticles on *Escherichia coli* in the dark. Proteomics.

[103] Chen Z., Han S., Zheng P., Zhou D., Zhou S., Jia G. (2020). Effect of oral exposure to titanium dioxide nanoparticles on lipid metabolism in Sprague-Dawley rats. Nanoscale.

[104] Papazyan R., Sun Z., Kim Y.H., Titchenell P.M., Hill D.A., Lu W., Damle M., Wan M., Zhang Y., Briggs E.R. (2016). Physiological Suppression of Lipotoxic Liver Damage by Complementary Actions of HDAC3 and SCAP/SREBP. Cell Metab..

[105] Kumar P., Salve R., Gajbhiye K.R., Gajbhiye V., Virendra G., Kavita R.G., Seungpyo H. (2022). Stimuli-Responsive Nanocarriers. Recent Advances in Tailor-Made Therapeutics: Chapter 1—An Overview of Stimuli-Responsive Nanocarriers: State of the Art.

[106] Dobrovolskaia M.A., Aggarwal P., Hall J.B., McNeil S.E. (2008). Preclinical Studies To Understand Nanoparticle Interaction with the Immune System and Its Potential Effects on Nanoparticle Biodistribution. Mol. Pharm..

[107] Dobrovolskaia M.A. (2015). Pre-clinical immunotoxicity studies of nanotechnology-formulated drugs: Challenges, considerations and strategy. J. Control. Release.

[108] Jiang W., Wang Y., Wargo J.A., Lang F.F., Kim B.Y.S. (2021). Considerations for designing preclinical cancer immune nanomedicine studies. Nat. Nanotechnol..

